# Effects of Thermal Variables of Solidification on the Microstructure, Hardness, and Microhardness of Cu-Al-Ni-Fe Alloys

**DOI:** 10.3390/ma12081267

**Published:** 2019-04-18

**Authors:** Maurício Silva Nascimento, Givanildo Alves dos Santos, Rogério Teram, Vinícius Torres dos Santos, Márcio Rodrigues da Silva, Antonio Augusto Couto

**Affiliations:** 1 Department of Mechanics, Federal Institute of Education, Science and Technology of São Paulo, 01109-010 São Paulo, Brazil; givanildo@ifsp.edu.br (G.A.d.S.); rogerioteram@ifsp.edu.br (R.T.); 2 Salvador Arena Foundation Educational Center, 09850-550 São Bernardo do Campo, Brazil; vinicius.santos@termomecanica.com.br (V.T.d.S.); marcio.rdrgs.slv@gmail.com (M.R.d.S.); 3 Department of Research and Development, Termomecanica São Paulo S.A., 09612-000 São Bernardo do Campo, Brazil; 4 Nuclear and Energy Research Institute, Center for Materials Science and Technology, IPEN, 05508-000 São Paulo, Brazil; aacouto@ipen.br; 5 Department of Engineering, Mackenzie Presbyterian University, UPM, 01302-907 São Paulo, Brazil

**Keywords:** solidification thermal parameters, Cu-Al-Ni-Fe bronze alloys, hardness, microhardness, specific intermetallics

## Abstract

Aluminum bronze is a complex group of copper-based alloys that may include up to 14% aluminum, but lower amounts of nickel and iron are also added, as they differently affect alloy characteristics such as strength, ductility, and corrosion resistance. The phase transformations of nickel aluminum–bronze alloys have been the subject of many studies due to the formations of intermetallics promoted by slow cooling. In the present investigation, quaternary systems of aluminum bronze alloys, specifically Cu–10wt%Al–5wt%Ni–5wt%Fe (hypoeutectoid bronze) and Cu–14wt%Al–5wt%Ni–5wi%Fe (hypereutectoid bronze), were directionally solidified upward under transient heat flow conditions. The experimental parameters measured included solidification thermal parameters such as the tip growth rate (V_L_) and cooling rate (T_R_), optical microscopy, scanning electron microscopy (SEM) analysis, hardness, and microhardness. We observed that the hardness and microhardness values vary according to the thermal parameters and solidification. We also observed that the Cu–14wt%Al–5wt%Ni–5wi%Fe alloy presented higher hardness values and a more refined structure than the Cu–10wt%Al–5wt%Ni–5wt%Fe alloy. SEM analysis proved the presence of specific intermetallics for each alloy.

## 1. Introduction

Cast copper alloys are used in applications that require metals with superior corrosion resistance, high electrical and thermal conductivity, good surface quality for bearings, and other special properties. Among the full range of copper alloys, aluminum bronzes are the best available material for fulfilling these requirements [1,2]. Aluminum bronzes are copper-based alloys that may include up to 14% aluminum, but lower amount of nickel and iron are also added to produce different alloy strength, ductility, and corrosion resistance [3,4,5,6]. In the maritime field, nickel aluminum–bronze alloys are known as “propeller bronze”, representing their application in the manufacturing of propellers of ships and submarines [7].

In the Cu-Al-Ni-Fe alloys, the aluminum component is the main alloying element, with a content normally varying between 8% and 13%. Greater contents are used for obtaining high hardness and reduce the ductility of the alloy. However, high levels of aluminum provide the appearance of γ2 phase, which is detrimental to its mechanical resistance and corrosion. Some elements such as Ni and Fe combine with Al to form complex phases called к, avoiding the emergence of the γ2. Nickel is added in amounts ranging from 1% to 7% and its presence improves corrosion resistance, increases mechanical strength, and contributes to increased erosion resistance in environments with high water flow velocity. Iron is present in nickel aluminum–bronze to refine the structure and increase the toughness. The low solubility of iron at low temperatures in these alloys is the main reason for the appearance of precipitates rich in iron, which can be combined to produce the required mechanical properties [6].

**Figure 1:**
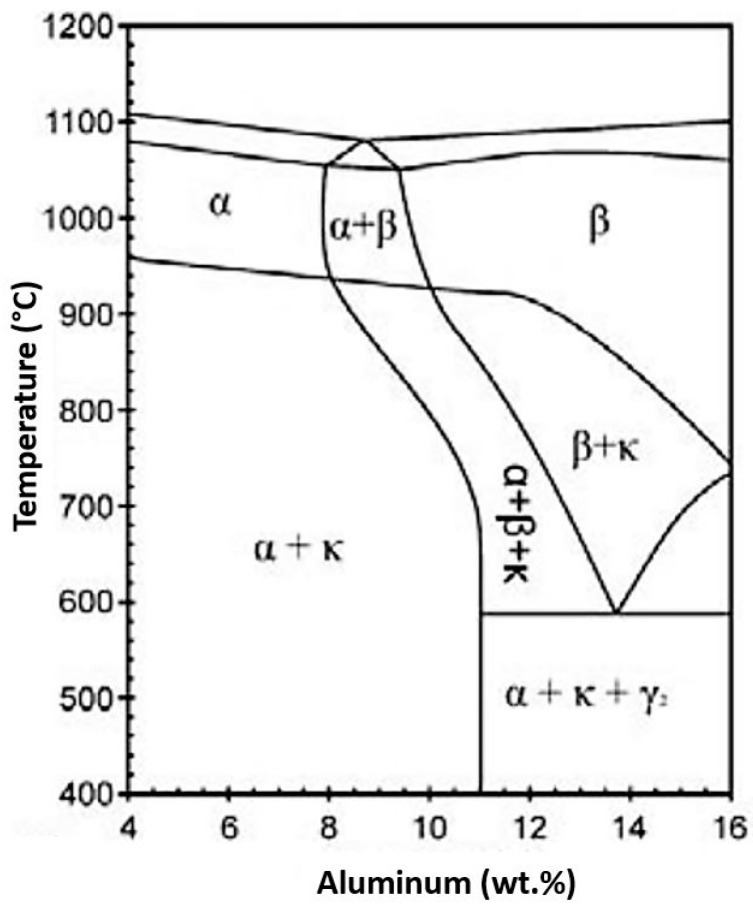
Phase diagram of the Cu-Al system with addition of 5 wt% nickel and 5 wt% iron [12].

The phase transformations of aluminum–bronze have been the subject of many studies due to the formations of intermetallics promoted by slow cooling [4,8,9,10,11]. The phase diagram of the Cu-Al system shows the different microstructures that arise in the cooling of the investigated alloys (Figure 1).

**Figure 2:**
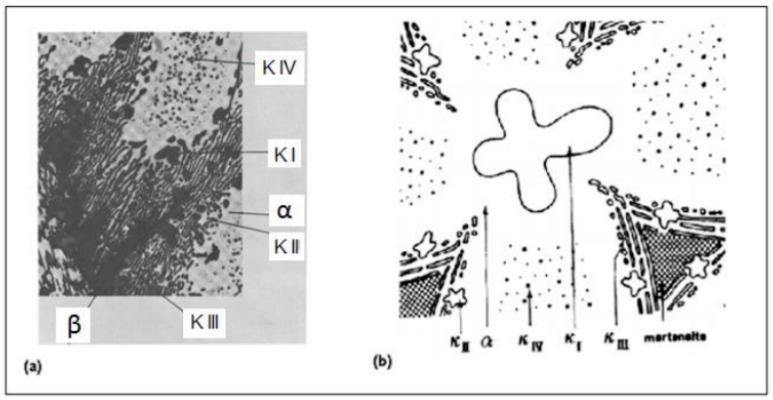
Distribution of the different phases and intermetallic components of the nickel aluminum–bronze cooled slowly: (**a**) optical microscopy [4] and (**b**) schematic representation [8].

The β phase is a solid solution phase at high temperatures in nickel aluminum–bronze and presents disordered BCC (body-centered cubic) crystalline structure. The β phase is present mainly at high temperatures and is considered the first solid generated in the transformation of the liquid state to the solid state; later, part of the β phase becomes the α phase [7]. The α phase represents a solid equilibrium solution or matrix with a FCC (face-centered cubic) crystalline structure. The α phase is formed from the β phase around 1030 °C and exhibits a Widmastätten structure [6,9,13]. In addition to the α phase, the aluminum–bronze alloy also exhibits a β phase that originates from three main types of intermetallics present in these alloys, labeled к, which is formed via slow cooling: Kappa II (кII), Kappa III (кIII), and Kappa IV (кIV), shown in Figure 2 [4,6,7,8,9,10,11,13].

We aimed to study the microstructure resulting from Cu-10wt%Al-5wt%Ni-5wt%Fe (hypoeutectoid bronze, cited as Cu10Al alloy) and Cu-14wt%Al-5wt%Ni-5wi%Fe (hypereutectoid bronze, cited as Cu14Al alloy) alloys after undergoing a directional solidification process. Directional solidification allows different microstructures to be obtained in the length of the molten ingot, influencing the alloy properties. The effects of the manufacturing processes on the microstructure and properties of engineering materials have been highlighted in various studies [14,15,16,17,18,19,20]. Thermal parameters of solidification, as tip growth rate (V_L_) and cooling rate (T_R_), were correlated with hardness and microhardness values for both alloys studied. Optical microscopy and scanning electron microcopy (SEM) images were obtained from various positions in the ingot for both alloys.

## 2. Materials and Methods

**Figure 3:**
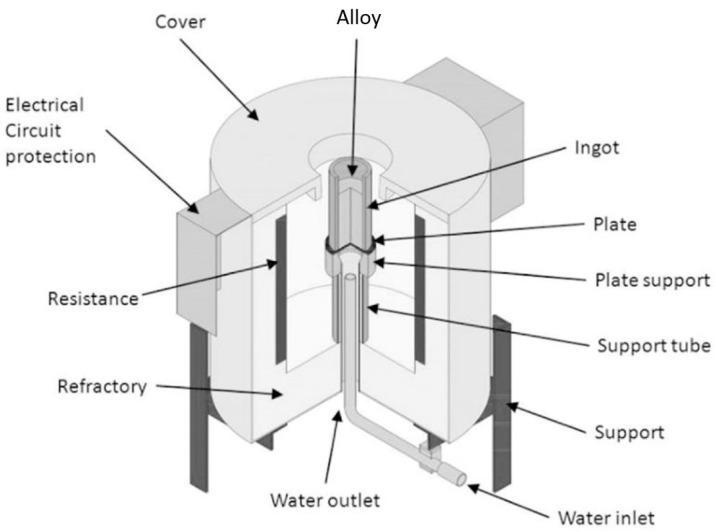
Schematic illustration of the upward unidirectional solidification furnace [14].

The directional solidification apparatus has a cylindrical shape (Figure 3), covered with refractory bricks and externally coated with steel plate. The heat required to keep the liquid metal heated before the cooling process was created by electrical resistors controlled with an external control panel. Two support tubes supported the ingot, the outer one being composed of SAE 1020 steel, and the internal tube was stainless steel AISI 304. Refractory cement was placed between these two tubes to increase the insulation of the internal space of the furnace. A tube inside the two support tubes directed the water jet into a plate responsible for the removal of heat from the molten metal. This plate was composed of SAE 1020 steel and was 5 mm thick. The upper surface of the sheet, which remained in contact with the liquid metal, was sanded with 1200 mesh sandpaper. The ingot mold was composed of stainless steel AISI 304, with a height of 160 mm and internal and external diameters of 60 and 76 mm, respectively. For the acquisition of temperature data, type K thermocouples were used, with distances of 4, 8, 12, 16, 35, 53, and 73 mm relative to the position of the upper surface of the heat exchange plate. These thermocouples were connected to National Instruments NI 9212 (National Instruments, Debrecen, Hungary) and NI cDAQ 9171 data acquisition devices (National Instruments, Debrecen, Hungary), responsible for sending the collected data to a computer via a USB cable. The temperature data obtained by the thermocouples were provided at the frequency of one per second.

The alloys were cast in a Fortelab muffle-type electric furnace (Fortelab, São Carlos, SP, Brazil) in a Salamander SIC AS2 graphite crucible. The chemical composition of the alloys was analyzed using X-ray spectrometry (XRS) using a Panalitycal Magix Fast Spectrometer (Panalitycal, Almelo, The Netherlands) (Table 1). The alloys were heated to temperatures above their liquid temperature. After this, the crucible was removed from the furnace and the liquid metal was poured into the ingot mold in the unidirectional solidification furnace. Cooling of the liquid metal inside the ingot started when the water jet was connected at a flow rate of 18 L/min.

The tip growth rate (V_L_) was calculated by deriving the function P = f(t). This function is the relationship between the position of the thermocouple (P) and the time interval between the start of the alloy cooling and the time at which the liquidus temperature (T_L_) is observed in each thermocouple. With this, V_L_ corresponds to the velocity of the solidification front passage in each thermocouple. The cooling rate (T_R_) values for each position on the thermocouple were obtained experimentally from the temperature variation values as a function of time, at a temperature before and after the *liquidus* temperature (ΔT/Δt). For metallographic analysis, samples of cross-sections of the molten ingot were selected. The analyzed surfaces of the samples were selected from different positions (P) in relation to the heat exchange surface. These distances were 4, 8, 12, 16, 26, 35 and 53 mm. Each sample was embedded in Bakelite, sanded with sands of different granulations, and polished with 3–6 μm diamond paste. The etchant used to reveal the microstructure consisted of a solution of 10.7% HCl, 3.4% Fe_3_Cl, and 85.9% distilled water. The reaction time was 25 s. A Zeiss AxioVert A1 microscope (Carl Zeiss, Gottingen, Germany) was used to obtain optical images of the microstructure. Samples were analyzed by scanning electron microscopy (SEM) using Phenom Pro X and Jeol JSM 6510 equipment (Jeol, Tokyo, Japan) for checking the phases and intermetallics morphology. The mechanical characteristics were evaluated by the hardness test, according to ASTM E10-2012 [21] standard in a Wilson UH-930 hardness tester (Boehler, Lake Bluff, IL, USA) using a load of 62.5 kgf and a sphere 2.5 mm in diameter. The hardness test was performed at five points of each position on the thermocouple. The microhardness was tested according to ASTM E92-2003 [22] standard in a Boehler VH1102 microhardness tester (Boehler, Lake Bluff, IL, USA) at five different points of each position on the thermocouple using force of 1 kgf.

## 3. Results and Discussion

**Figure 4:**
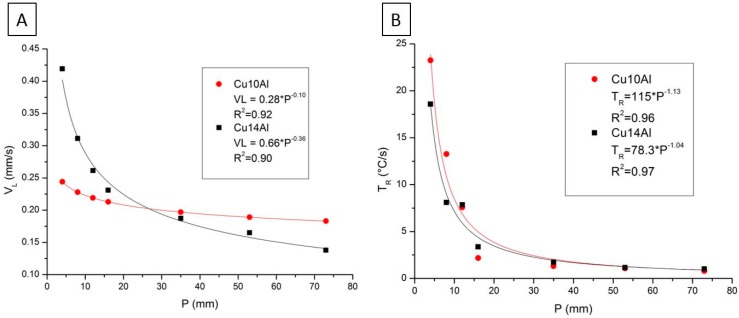
Solidification thermal parameters: (**A**) correlation between tip growth rate (V_L_) and distance from heat extraction surface (P); and (**B**) correlation between cooling rate (T_R_) and distance from heat extraction surface (P). The error bars represent the standard deviation of the measurements obtained.

Figure 4 presents the thermal parameters V_L_ and T_R_ experimentally obtained as a function of the distance to the heat exchange surface (P). For both alloys, V_L_ values decreased with higher P values. The Cu14Al alloy, which has a higher amount of Al in its composition, had higher initial V_L_ values than the Cu10Al alloy (Figure 4A). The values of T_R_, similar to V_L_, decreased as the distance from the heat exchange surface (P) increased. We observed that the Cu10Al alloy had values slightly larger than those for the Cu14Al alloy (Figure 4B). Analyzing the results obtained for both alloys, we observed that the Al content influences the values of V_L_ and T_R_.

**Figure 5:**
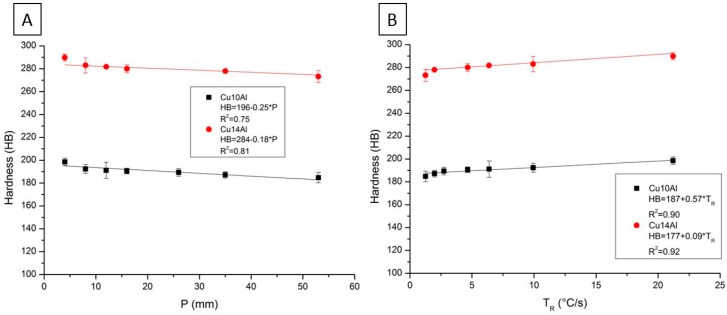
(**A**) Correlation between hardness (HB) and distance from heat extraction surface (P); and (**B**) correlation between HB and cooling rate (T_R_). The error bars represent the standard deviation of the measurements obtained.

The data obtained in the hardness test are presented in Figure 5. The experimental equations that correlate the hardness values (HB) with the distance of the heat exchange surface (P) values and with the values of T_R_ in the graphs were obtained by the least square method using Origin software. The linear fit of the data suggests that the hardness values (HB) decrease with increasing distance of the heat exchange surface (P). For T_R_, the adjustment indicates the opposite: the values of hardness increase with the increase in T_R_. This is important because it shows that it is possible to predict the hardness performance of both alloys by changing the cooling conditions. Comparing the two alloys studied, the Cu14Al alloy has higher hardness values than the Cu10Al alloy. This suggests that the increase in Al content influences this property. The linear adjustment also suggests that there are maximum hardness values. If P = 0 mm, we can define these values. For the Cu10Al and Cu14Al alloys, the values were 196 and 284 HB, respectively.

**Figure 6:**
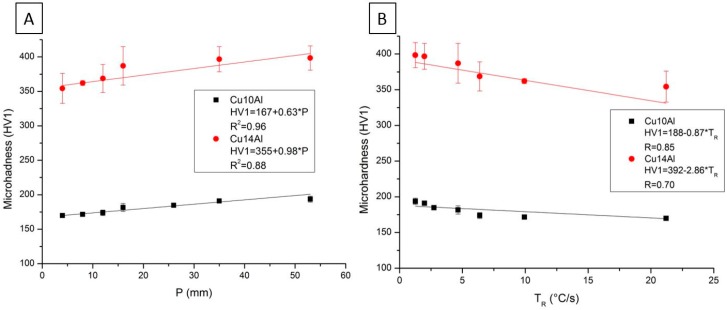
(**A**) Correlation between microhardness (HV1) and distance from heat extraction surface (P); and (**B**) correlation between microhardness (HV1) and cooling rate (T_R_). The error bars represent the standard deviation of the measurements obtained.

The data obtained in the microhardness test are presented in Figure 6. The experimental equations correlate the microhardness values (HV1) with the distance of the heat exchange surface (P). The values of T_R_ presented in the graphs were obtained by the least square method using Origin software. The linear fit of the data suggests that HV1 increases with the increase in the distance of the heat exchange surface (P). For T_R_, the adjustment indicates the opposite: the values of hardness decrease with the increase in T_R_.

**Figure 7:**
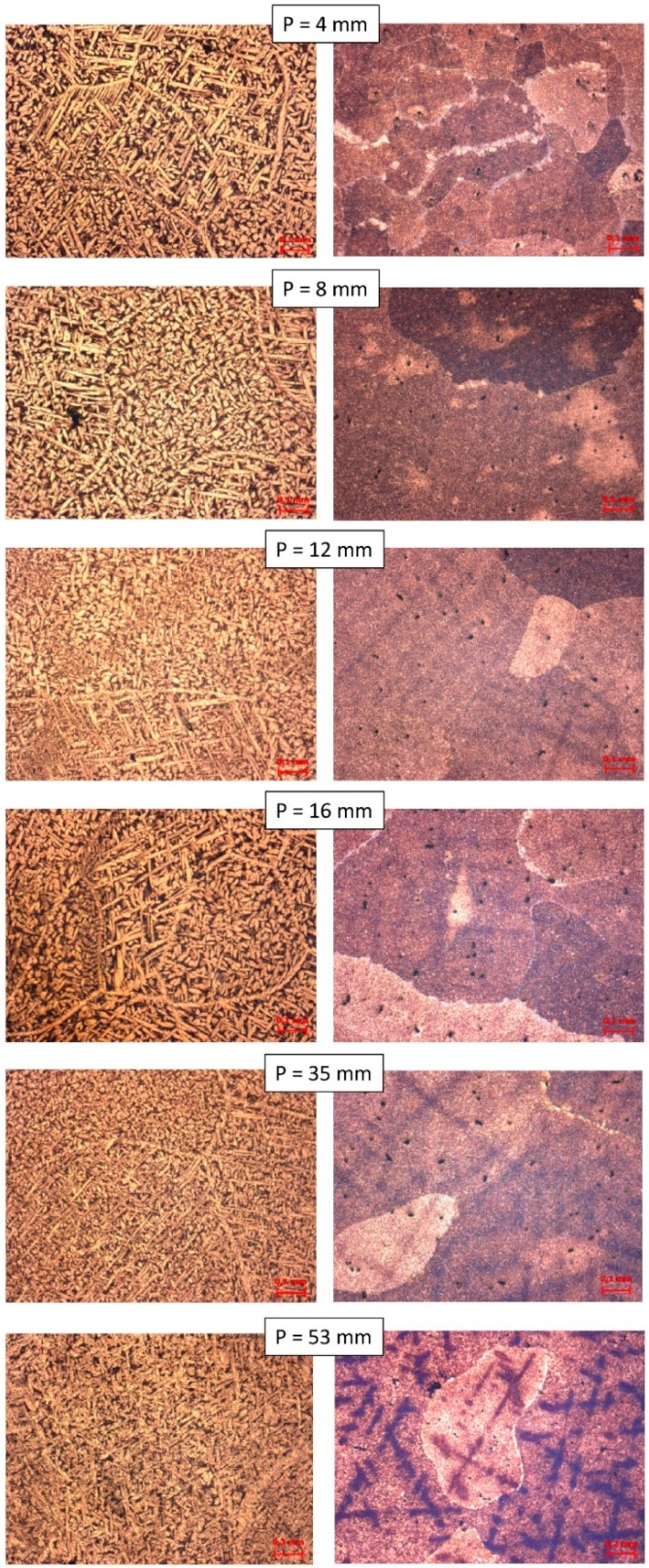
Micrograph of the various samples taken from different positions (P) as a function of the heat exchange surface: (**left**) Cu10Al alloy; and (**right**) Cu14Al alloy.

Figure 7 depicts the transverse micrographs of the two alloys studied at positions 4, 8, 12, 16, 35, and 53 mm with respect to the heat extraction surface (P). Comparing both alloys, the Cu10Al alloy presents the α phase in Widmastätten morphology, whereas the Cu14Al alloy presents a diffuse morphology with small microstructures inside the grain. At the position P = 4 mm, we observed that the grains of the Cu14Al alloy have smaller dimensions than for the Cu10Al alloy. At position P = 53 mm, the dendritic arms were observed in dark color for the Cu14Al alloy. The dendritic arms being in positions of higher values of P and not in smaller values, show that the grain size increases as the value of P increases.

**Figure 8:**
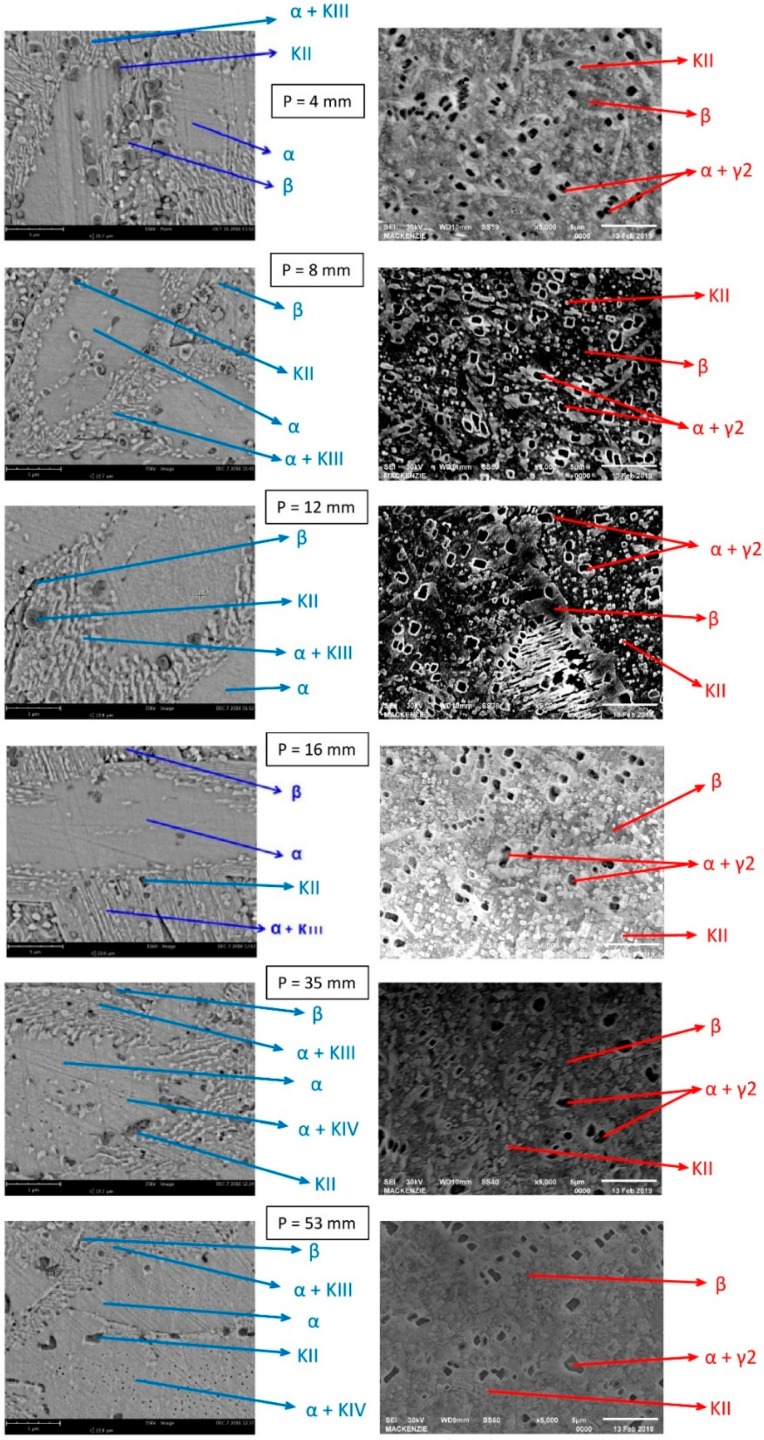
Scanning electron microscopy (SEM) analysis of the various samples taken from different positions (P) as a function of the heat exchange surface: (**left**) Cu10Al alloy; and (**right**) Cu14Al alloy.

The images obtained by SEM for both alloys are shown in Figure 8. Hasan et al. [8] studied the morphology, crystallography, and composition of the phases present in Cu10Al alloy, determining the characteristics of each phase. Jahanafrooz et al. [9] studied the mechanism of phase formation in Cu10Al alloy during solidification. Pisarek [11] proposed a crystallization model for Cu-Al-Ni-Fe alloys. The microconstituents in the SEM images obtained in this work were identified based on the similarity of the SEM images presented by the authors mentioned above. We observed that the Cu14Al alloy had a larger number of microconstituents. The Cu10Al alloy more prominently presents the α phase.

The Cu14Al alloy had higher hardness values, a structure with smaller grains, and more microconstituents evidenced by the SEM analysis than the Cu10Al alloy, suggesting that the higher Al content influences these properties. It should be noted that the Cu14Al alloy presents the γ2 phase, characteristic of the high aluminum content in the alloy. This phase is detrimental because it reduces the performance of the alloy for corrosion resistance. The fact that this alloy has in its composition Fe and Ni contents, this phase appears in smaller quantity, since these elements bind to Al forming the microconstituents к mentioned above. It is also observed the appearance of the retained beta phase caused by the high rate of cooling. This phase is martensitic giving higher hardness values for the alloy.

## 4. Conclusions

The values of the solidification thermal parameters V_L_ and T_R_ decrease for larger distances from the heat exchange surface. The Cu14Al alloy, which has the highest amount of Al in its composition, has higher initial V_L_ higher values than the Cu10Al alloy. The linear fit of the data suggests that the hardness values (HB) decrease with increasing distance from the heat exchange surface (P). For T_R_, the adjustment indicates the opposite: the values of hardness increase with increasing T_R_ values. The linear fit of the data also suggests that the microhardness values (HV1) increase with increasing distance from the heat exchange surface (P). For T_R_, the adjustment indicates the opposite: the values of hardness decrease with increasing T_R_.

Comparing the transverse optical micrographs for both alloys, the Cu10Al alloy presents the α phase in Widmastätten morphology, whereas the Cu14Al alloy presents a diffuse morphology with small microstructures inside the grain. At position P = 4 mm, we observed that the grains of the Cu14Al alloy were smaller than those in the Cu10Al alloy. At position P = 53 mm, the dendritic arms were observed to have a dark color for the Cu14Al alloy. The ability to observe the dendritic arms at positions of higher value of P and not in smaller values shows that the size of the grain increases as the value of P increases.

In the SEM images for both alloys, we observed that the Cu14Al alloy has more microconstituents. The Cu10Al alloy presented the most prominent α phase. The Cu14Al alloy has higher hardness values, a structure with smaller grains, and more microconstituents, as evidenced by the SEM analysis, than the Cu10Al alloy, suggesting that the higher Al content influences these properties.

## Figures and Tables

**Table 1:** Chemical composition of ingots in weight%.

Alloy	Al	Ni	Fe	Others	Cu
Cu–10wt%Al–5wt%Ni–5wt%Fe	10.79	4.42	3.67	0.051	Remaining
Cu–14wt%Al–5wt%Ni-5wt%Fe	14.23	5.44	5.39	0.340	Remaining
